# Induced pluripotent stem cell (iPSC) modeling validates reduced GBE1 enzyme activity due to a novel variant, p.Ile694Asn, found in a patient with suspected glycogen storage disease IV

**DOI:** 10.1016/j.ymgmr.2024.101069

**Published:** 2024-03-14

**Authors:** Chie Naito, Karis Kosar, Eriko Kishimoto, Loren Pena, Yilun Huang, Kaili Hao, Anas Bernieh, Jennifer Kasten, Chet Villa, Priya Kishnani, Bali Deeksha, Mingxia Gu, Akihiro Asai

**Affiliations:** a Cincinnati Children's Hospital Medical Center, Cincinnati, OH, USA; b Department of Pediatrics, University of Cincinnati College of Medicine, Cincinnati, OH, USA; c Department of Pediatrics, Division of Medical Genetics, Duke Health, Durham, NC, USA

**Keywords:** Glycogen storage disease IV (GSD4), Glycogen branching enzyme 1 (GBE1), iPSCs (induced pluripotent stem cells), GBE1 enzyme activity, Hepatocyte differentiation, Cardiomyocyte differentiation

## Abstract

**Background:**

Glycogen Storage disease type 4 (GSD4), a rare disease caused by glycogen branching enzyme 1 (GBE1) deficiency, affects multiple organ systems including the muscles, liver, heart, and central nervous system. Here we report a GSD4 patient, who presented with severe hepatosplenomegaly and cardiac ventricular hypertrophy. *GBE1* sequencing identified two variants: a known pathogenic missense variant, c.1544G>A (p.Arg515His), and a missense variant of unknown significance (VUS), c.2081T>A (p. Ile694Asn). As a liver transplant alone can exacerbate heart dysfunction in GSD4 patients, a precise diagnosis is essential for liver transplant indication. To characterize the disease-causing variant, we modeled patient-specific GBE1 deficiency using CRISPR/Cas9 genome-edited induced pluripotent stem cells (iPSCs).

**Methods:**

iPSCs from a healthy donor (iPSC-WT) were genome-edited by CRISPR/Cas9 to induce homozygous p.Ile694Asn in *GBE1* (iPSC-GBE1-I694N) and differentiated into hepatocytes (iHep) or cardiomyocytes (iCM). GBE1 enzyme activity was measured, and PAS-D staining was performed to analyze polyglucosan deposition in these cells.

**Results:**

iPSC^GBE1-I694N^ differentiated into iHep and iCM exhibited reduced GBE1 protein level and enzyme activity in both cell types compared to iPSC^wt^. Both iHep^GBE1-I694N^ and iCM^GBE1-I694N^ showed polyglucosan deposits correlating to the histologic patterns of the patient's biopsies.

**Conclusions:**

iPSC-based disease modeling supported a loss of function effect of p.Ile694Asn in *GBE1*. The modeling of GBE1 enzyme deficiency in iHep and iCM cell lines had multi-organ findings, demonstrating iPSC-based modeling usefulness in elucidating the effects of novel VUS in ultra-rare diseases.

## 1 Introduction

Aberrant glycogen storage and metabolism are hallmarks of glycogen storage diseases (GSD), a set of rare metabolic disorders that affect 1 in every 10,000–43,000 births [1,2]. Accounting for less than 3% of all GSD diagnoses, glycogen storage disease IV (GSD4) is an autosomal recessive disorder caused by glycogen branching enzyme 1 (GBE1) deficiency, resulting in the accumulation of polyglucosan, an abnormal glycogen, in affected tissues, such as the liver, heart, skeletal muscle, and brain to varying degrees [1,[3], [4], [5], [6]]. Due to the variable activities of GBE1 in different cell types, clinical manifestations of GSD4 differ among patients significantly [2,3]. In the progressive hepatic GSD4 subtype, children present with failure to thrive, portal hypertension, hepatosplenomegaly, and progress at variable speeds to cirrhosis and liver failure within the first 18 months of life [5,7,8]. Once in liver failure, liver transplantation is the only treatment for these patients to survive [4,5,9]. However, despite transplantation, the prognosis remains poor due to polyglucosan accumulation in cardiomyocytes, contributing to the development of cardiomyopathy. The mortality risk is significant in 30–40% of post-transplant patients, largely due to these extrahepatic GSD4 manifestations, indicating the profound importance of a case-by-case approach for the indication of liver transplant [5,[9], [10], [11]]. Therefore, it is crucial to consider the complexity and severity of GSD4, which extends beyond a singular organ, warranting comprehensive multi-organ consideration for effective treatment, planning, and evaluation of GSD4 manifestations during liver transplant evaluation [8,12].

**Fig. 1:**
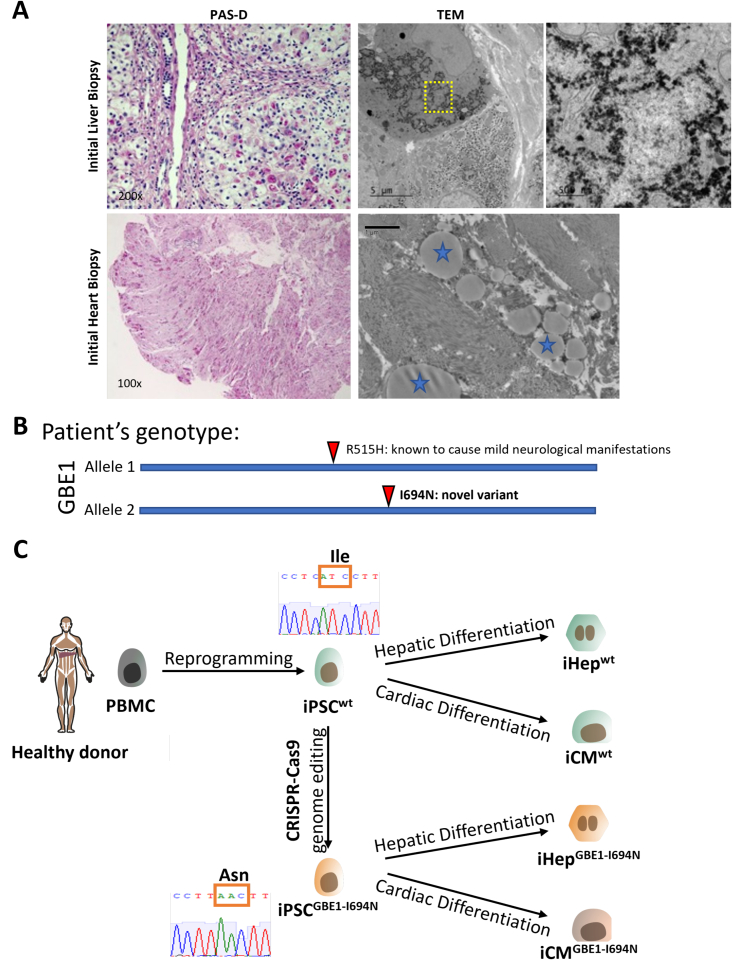
GSD4 patient presents in clinic with a novel *GBE1* variant. A) Patient biopsies from both the heart and liver showed typical patterns of GSD4 with polyglucosan accumulation visible through PAS-D positive hepatocyte intracytoplasmic inclusions and filamentous nonbranching cytoplasmic aggregates visible (marked with blue stars) through electron microscopy in both hepatocytes and cardiomyocytes. PAS-D images were taken on scales of X200 microscope setting for liver biopsy and x100 for the heart. B) Schematic representation of patient's *GBE1* genotype. Two variants identified *GBE1*: p. Arg515His, known to cause mild late-onset neurological manifestation and p.Ile694Asn, a novel missense variant with unknown clinical significance. C) Scheme for generating iPSCs containing the novel p.Ile694Asn *GBE1* variant. iPSCs generated from a healthy donor were genome-edited by CRISPR/Cas9 to be homozygous for the novel variant. Both WT control and genome-edited iPSCs were then differentiated into either hepatocytes or cardiomyocytes to study the effects of the novel variant on GBE1 function.

Here, we present a case of a one-year-old boy with a suspected GSD4 diagnosis, who presented with severe hepatosplenomegaly, cardiac ventricular hypertrophy, and failure to thrive. Liver biopsy showed typical GSD4 patterns with PAS-positive and diastase-resistant cytoplasmic inclusions in hepatocytes and filamentous nonbranching cytoplasmic aggregates visible with electron microscopy (Fig. 1A). Subsequent heart biopsy showed evidence of extrahepatic GSD4 manifestation, with most cardiomyocytes showing cytoplasmic, perinuclear polyglucosan bodies (Fig. 1A). In addition, the GBE1 enzyme activity in the liver biopsy tissue was reduced at 4 μmol/min/gram-tissue (normal range: 85±31 μmol/min/gram-tissue). Targeted *GBE1* sequencing identified two variants: a known pathogenic missense variant, c.1544G>A (p. Arg515His), [7,13,14], and a missense variant, c.2081T>A (p.Ile694Asn) previously reported as a variant of unknown significance (VUS) [15,16] (Fig. 1B). At the time of presentation, these reports for p.Ile694Asn were not available, making the variant novel and this genetic test inconclusive. The parents' heterozygous genotype for either variant confirmed *trans* configuration for the *GBE1* variants. In the homozygous state, the variant c.1544G>A (p.Arg515His) is associated with adult polyglucosan body disease (APBD), characterized by dysfunction of the central and peripheral nervous systems without overt liver involvement [7,13,14]. Given the reduction in GBE1 enzyme activity and that the proband's phenotype did not correlate with APBD, we suspected that c.2081T>A (p.Ile694Asn) led to loss of GBE1 function resulting in the proband's disease phenotype.

Determining the novel VUS's impact on the liver and heart is imperative, as genetic confirmation of disease-causing variants informs clinicians in transplant teams. In cases of concurrent liver and heart involvement, assessing the disease course is crucial, as the timing of one organ's transplant affects the other's functionality. Given the complexities in cases with numerous unknown variables, establishing a GSD4 subtype diagnosis and determining the VUS's effect significantly influences clinical decision-making. Once a genotype-phenotype correlation is identified, disease course predictions at the early stage become feasible. To this end, we aimed to model patient-specific GBE1 deficiency *in vitro* using CRISPR/Cas9-edited iPSCs.

Human induced pluripotent stem cells (iPSCs) offer a tailored method for studying disease, as CRISPR/Cas9 editing can be utilized to express novel variants to understand their effect on the disease phenotype [17]. These edited iPSCs can then be differentiated into cell types of interest, such as hepatocytes and cardiomyocytes [18,19], and used to explore the role of unknown variants in the tissue. Thus, utilizing CRISPR/Cas9 technology we generated iPSCs with the proband's VUS and compared phenotypes to the parental wild-type iPSCs after differentiating into either hepatocytes or cardiomyocytes (Fig. 1C). These findings were critically important as they offered a unique insight into the tissue phenotype, in addition to demonstrating the application of iPSC-based modeling to help predict multiple organ involvement in ultrarare disease cases with VUS.

## 2 Materials and methods

**Culturing Human iPSCs and CRISPR/Cas9 editing of GBE1:** Human specimens were obtained from patients according to ethically approved protocols (Study ID: 2014–6705 for the Institutional Review Board of Cincinnati Children's Hospital Medical Center). Human iPSCs were edited by CRISP/Cas9 in a method described previously [20]. Briefly, control iPSCs (clone code: 1383D6), characterized for pluripotency, karyotyped, and provided by Kyoto University [21], underwent CRISPR/CAS9 genome-editing to introduce homozygous *GBE1* VUS (c.2081T>A (p.Ile694Asn) by selecting candidate single-guide RNA (sgRNA) from CRISPOR (http://crispor.org/), with exceptionally high scores for specificity [22]. A single-stranded oligonucleotide-DNA (ssODN) was designed including the desired nucleotide alteration and to create new restriction enzyme sites for genotyping. One million single-cell ACCUTASE™ (Stem Cell Tech #07920) prepared iPSCs were nucleofected with sgRNA, purified Cas9 protein, and 2.5 μg of ssODN using program CA137 (Lonza). Forty-eight hours post-transfection, cells were propagated, and correctly edited clones were selected based on the loss of the restriction enzyme sites on both alleles. Sanger sequencing further confirmed the identification of bi-allelic single nucleotide alteration. Cell clones that remained unedited after this process were used as isogenic parental controls.

**Differentiation of iPSCs to hepatocytes (iHeps):** Both iPSC lines were differentiated into hepatocytes (iHeps) using a modified method previously described [23]. Briefly, iPSCs were ACCUTASE™ dissociated and plated onto iMatrix-511 SILK (Iwai North America #N-892021) coated 48-well cell culture plates with RPMI 1640 (Thermo Fisher Scientific #22400105) containing 2% B27 (Thermo Fisher Scientific #17504044), 50 ng/mL Wnt3a (R&D Systems #5036-WN-MTO), 100 μg/mL Activin (Shenandoah #800–01), and Y-27632 (StemCell Technologies #72304). The following day, the medium was replaced with RPMI with 2% B27, 50 ng/mL Wnt3a, 100 μg/mL Activin, and 0.5 mM sodium butyrate (Sigma #B5887-1G) for 3 days, and without sodium butyrate for 3 days. For hepatic specification, cells were further treated with 10 ng/mL fibroblast growth factor 2 (R&D Systems #233-FB), 20 ng/mL bone morphogenetic protein 4 (R&D Systems #314-BP), and 1% MEM Non-Essential Amino Acids Solution (Thermo Fisher Scientific #11140050) for 3 days. Cells were then cultured in Hepatocyte Culture Medium (HCM) with HCM Bullet Kit (Lonza #CC-3198) omitting human epidermal growth factor (hEGF), with the addition of 10 ng/mL recombinant hepatocyte growth factor (HGF) (Pepro Tech #100–39), 100 nM dexamethasone, and 5% of FBS (ThermoFisher # 10437028) for 12 days, with medium changes every 3 days. A day before experiments, HGF and FBS were removed. To determine successful iHep differentiation, medium albumin concentration was measured using the Human Albumin ELISA Kit (Abcam #ab1798870).

**Differentiation of iPSCs to cardiomyocytes (iCM) and flow cytometry:** iCMs differentiation was performed following a previously published method [24]. Briefly, both iPSC lines were cultured in StemMACS™ iPS-Brew medium (Miltenyi #130104368) in 0.1 mg/mL Matrigel-coated 6-well plates. When reaching ≥80% confluence, the medium was changed to RPMI 1640 (Thermo Fisher Scientific #72400146) supplemented with B27 minus insulin (Thermo Fisher Scientific #A18956001) (iCM differentiation medium) and 6 μM CHIR99021 (Selleck Chemicals, S2924). After 2 days, the iCM differentiation medium was supplemented with 5 μM IWP2 (Selleck Chemicals #S7085) and changed every two days until day 8, when spontaneously beating cells appeared. On day 8, the medium was changed to RPMI 1640 with B27 plus insulin (Thermo Fisher Scientific #17504044) and replaced every 2 days until cells were collected.

Flow cytometry of iCMs was performed by modifying a previously published method [25]. iCMs were dissociated with 0.05% Trypsin-EDTA solution (Thermo Fisher Scientific #25300054) in culture medium for 7 min at room temperature and collected by centrifugations at 4 °C. Cells were fixed for 15 min on ice with 4% paraformaldehyde (PFA) in PBS (Santa Cruz #sc-281692) followed by permeabilization using 0.2% Triton X-100 (Sigma, T8787) in PBS. Cells were washed twice with 4% PFA in PBS, resuspended in a blocking solution containing 10% donkey serum (Jackson Lab #017000121) in PBS, followed by staining with anti-cardiac Troponin T antibody (Abcam #ab92546, 1:1000) for 45 min on ice. Stained cells were washed in PBS with 1% FBS (Thermo Fisher Scientific #26140079) and stained with secondary antibody for 30 min on ice. Stained cells were analyzed using the BD LSRII Flow cytometer. Data were analyzed using FlowJo software.

**Western blot analysis and GBE1 enzyme activity measurement:** Western blot analysis of GBE1 protein was performed using a method previously published [20]. Briefly, proteins were isolated using Cell Lysis Buffer (Cell Signaling Technology # 9803) with a proteinase and phosphatase inhibitor cocktail. Protein extracts were resolved by 4–12% SDS-PAGE, transferred to PVDF membranes and blocked in 5% skim milk. Membranes were incubated with primary antibodies against GBE1 (Proteintech #20313–1-AP, 1:1000) or beta-actin (Sigma #A3293, 1:1000) overnight at 4°C, washed, and incubated with horseradish peroxidase-conjugated secondary antibodies for 1  hour at room temperature. Chemiluminescence reagents were used to visualize the proteins, and images were captured using the Chemi-doc system (Bio-Rad).

Branching enzyme activity (GBE) was measured in snap-frozen liver tissues obtained by biopsies and frozen cell pellets obtained from iPSC lines. GBE activity in liver tissue was measured in a clinically standardized protocol (normal range: 85±31 μmol/min/gram-tissue). Heart tissues obtained by heart biopsy did not yield sufficient tissue amount to measure GBE enzyme activity. GBE activities in cell extracts were measured using the standard spectro-photometric method and phosphorylase as the indicating enzyme [26,27] and were compared between wild-type and GBE1-I694N after differentiation into iHep or iCM. The residual enzyme activity was assayed indirectly by measuring the amount of phosphate released using Roche phosphate reagent, and the activity was expressed as nmol/min/mg protein. Enzyme activity data was obtained for multiple replicates of iPSCs: *n* = 5 for each iHep and iCM from each cell line.

**Immunostaining and Periodic Acid-Schiff staining with diastase (PAS-D):** The immunostaining protocol was modified from previous reports [23]. Briefly, cells were fixed using 4% paraformaldehyde, 0.5% Triton X100 permeabilized, and blocked with 5% donkey serum. Primary antibody, HNF4a (Santa Cruz #sc-6556, 1:500), was incubated at 4°C overnight, secondary antibodies at room temperature for 1  hour, and Hoescht 33342 (Invitrogen #H3570, 1:1000) for 10  minutes at room temperature. Imaging was performed using an Olympus microscope and DP71 camera (Olympus, Center Valley, PA) and Zeiss LSM710 confocal microscope (San Diego, CA). To perform the PAS-D staining, formalin-fixed paraffin section of liver and heart samples and 4% paraformaldehyde-fixed cells were stained using the Periodic Acid Schiff Diastase Stain Kit (Abcam #ab287866).

## 3 Results

**Fig. 2:**
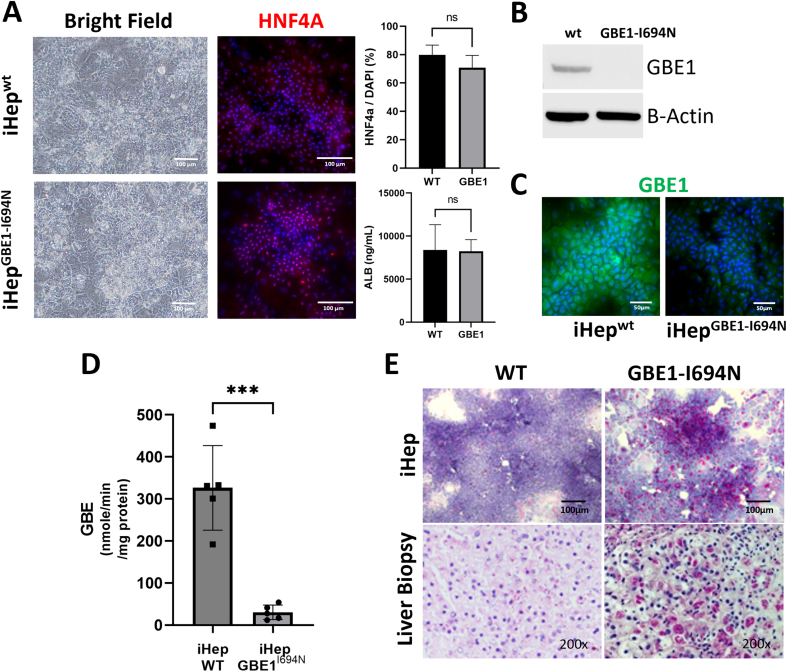
iPSCs containing novel GBE1 variant differentiate into hepatocytes and exhibit deficiency of GBE1 activity. A) Brightfield microscopy and immunofluorescent staining of hepatocyte nuclear factor-4 alpha (HNF4a: red, Hoechst nuclear stain: blue) revealed comparable patterns in iHep^wt^ and iHep^GBE1-I694N^. Additionally, there is no significant difference in albumin production in the culture supernatant (measured by ELISA) between iHep^wt^ and iHep^GBE1-I694N^. Sample size: *n* = 5 each for HNF4a staining, WT n = 5 and mutant *n* = 6 for albumin production. Graphs were generated using Prism GraphPad 9.5.0. *P* values were determined using the two-tailed *t*-test. *P* < 0.05 was considered statistically significant. B) Western blot analysis shows that GBE1 expression is comparable between iHep^wt^ and iHep^GBE1-I694N^. Beta-actin is used as a loading control. C) GBE1 enzyme assay showed significantly reduced activity in iHep^GBE1-I694N^ compared to iHep^wt^. n = 5 samples for both groups. ***: *p* = 0.001, using the two-tailed t-test. D) PAS-D staining revealed polyglucosan deposits in iHep^GBE1-I694N^, which correlates with the proband's liver biopsy. Both iHep^wt^ and healthy liver tissue were negative for PAS-D staining. Liver biopsy image was taken on a scale of x200.

**iPSCs containing *GBE1* missense VUS maintain their ability to differentiate into hepatocytes:** Control iPSCs generated from a healthy donor (iPSC^wt^) were CRISPR-Cas9 genome-edited to introduce the homozygous missense variant c.2081T>A (p.Ile694Asn) in *GBE1* (iPSC^GBE1-I694N^). The genotypes of both iPSC^wt^ and iPSC^GBE1-I694N^ were confirmed *via* Sanger sequencing of the c.2081 locus. iPSC^wt^ and iPSC^GBE1-I694N^ exhibited c.2081T (reference sequence) and c.2081A, respectively (Fig. 1C). Both lines were then differentiated into hepatocytes (iHep^wt^ and iHep^GBE1-I694N^), and morphological and functional evaluations were performed to determine whether iPSC^GBE1-I694N^ differentiate into functional hepatocytes. Using brightfield microscopy, no morphological differences were observed between iHep^wt^ and iHep^GBE1-I694N^ (Fig. 2A). Immunostaining for HNF4a, a hepatic differentiation marker, showed similar expression patterns in both iHep^wt^ and iHep^GBE1-I694N^, with a comparable HNF4a positive nuclei ratio (Fig. 2A). Albumin concentration of the culture medium measured *via* ELISA at the end of differentiation showed no significant difference in albumin production between iHep^wt^ and iHep^GBE1-I694N^ (Fig. 2A). Therefore, both iPSCs can comparably differentiate into hepatocytes.

**iHep**^**GBE1-I694N**^**have reduced GBE1 enzyme activity:** To evaluate if p.Ile694Asn disrupts *GBE1* expression in iHep^GBE1-I694^, we performed western blot analysis for GBE1. iHep^GBE1-I694N^ showed decreased GBE1 protein expression compared to iHep^wt^ (Fig. 2B). We then tested the enzyme activity of GBE1 in both iHeps. GBE1 activity in iHep^wt^ was 326.2 +/− 100.7 nmol/min/mg-protein, while GBE1 activity in iHep^GBE1-I694N^ was significantly reduced to 30.3 +/− 17.3 nmol/min/mg-protein (Fig. 2C). These findings indicate that homozygous p.Ile694Asn reduces GBE1 expression in hepatocytes, and this reduced protein level is the cause for the decrease in GBE1 function. We then performed a PAS-D stain on both iHeps to determine if the reduced GBE1 protein level and activity in iHep^GBE1-I694N^ induced polyglucosan deposition in cells. We found that iHep^GBE1-I694N^ had increased accumulation of polyglucosan compared to the iHep^wt^ as observed in the proband's liver biopsy. (Fig. 2D).

**Fig. 3:**
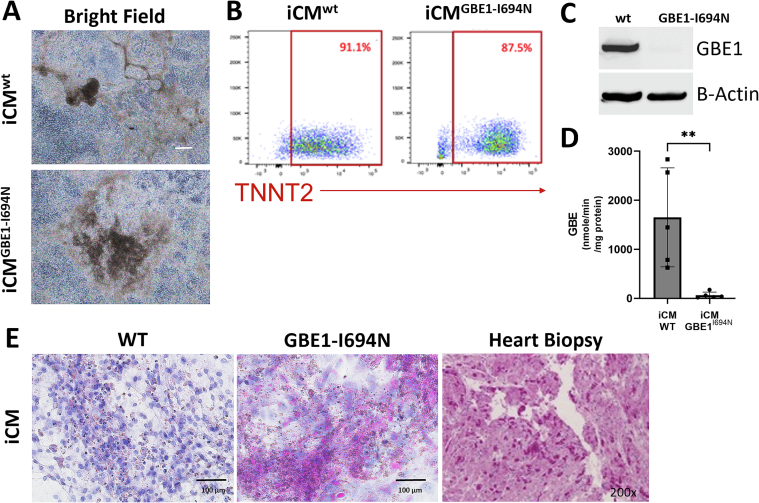
iPSCs containing novel GBE1 variant differentiate into cardiomyocytes and exhibit deficiency of GBE1 activity. A) Brightfield microscopy images reveal comparable morphology in iCM^wt^ and iCM^GBE1-I694N^. B) Using flow cytometry, iCM^wt^ and iCM^GBE1-I694N^ showed comparable expression levels of cardiomyocyte marker, TNNT2. C) Western blot analysis shows that GBE1 expression is comparable between iCM^wt^ and iCM^GBE1-I694N^. Beta-actin is used as a loading control. D) GBE1 enzyme assay shows significantly reduced activity in iCM^GBE1-I694N^ compared to iCM^wt^. n = 5 for both groups and ** *p* = 0.008, determined by the two-tailed t-test. E) PAS-D staining revealed polyglucosan deposits in iCM^GBE1-I694N^, correlating with the proband's initial heart biopsy. iCM^wt^ was negative for PAS-D. The heart biopsy image was taken on a scale of x200.

**iPSCs containing *GBE1* VUS maintain their ability to differentiate into cardiomyocytes:** After determining that iHep^GBE1-I694N^ modeled patient liver pathology, we aimed to determine whether p.Ile694Asn leads to a cardiomyocyte phenotype. First, we tested iPSCGBE1-I694N's ability to differentiate into cardiomyocytes compared to iPSC^wt^ (iCM^wt^ and iCM^GBE1-I694N^). Brightfield microscopy revealed that both iPSC^wt^ and iPSC^GBE1-I694N^ appear similar morphologically when differentiating to iCM^wt^ and iCM^GBE1-I694N^ (Fig. 3A), with both iCMs beating in culture once differentiation was completed. Using flow cytometry, both iCM^wt^ and iCM^GBE1-I694N^ were found to express troponin T (TNNT2), a cardiac-specific protein, comparably (Fig. 3B). Therefore, both iPSCs can comparably differentiate into cardiomyocytes.

**iCM**^**GBE1-I694N**^**have reduced GBE1enzyme activity:** Given the proband's heart biopsy was PAS-D positive, we also wanted to determine how GBE1 protein level and enzyme activity were affected in cardiomyocytes. We found that iCM^GBE1-I694N^ had reduced GBE1 protein levels (Fig. 3C) and enzyme activity of 62.3 +/− 64.4 nmol/min/mg-protein compared to 1652 +/− 1010 nmol/min/mg-protein in iCM^wt^ (Fig. 3D). These findings suggested that GBE1 protein levels were reduced in iCM^GBE1-I694N^ compared to iCM^wt^ which results in lessened GBE1 enzymatic activity in iCM^GBE1-I694N^. We also performed PAS-D staining on both iCMs to determine if decreased GBE1 activity in iCM^GBE1-I694N^ affected polyglucosan deposition. Using PAS-D staining we found that iCM^GBE1-I694N^ had increased polyglucosan accumulation compared to iCM^wt^, also indicating reduced GBE1 activity in iCM^GBE1-I694N^ (Fig. 3E). Additionally, PAS-D staining of iCM^GBE1-I694N^ correlated with the histological findings noted in the proband's heart biopsy (Fig. 3E).

**Fig. 4:**
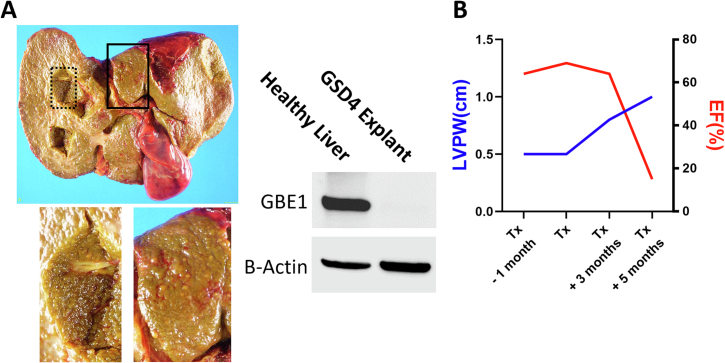
The clinical course and GBE1 protein expression in the liver explant. A) Liver explant of the proband showed extensive parenchymal fibrosis and cirrhosis. Higher magnification images from the black square and the dotted square show fibro-nodular parenchyma. Western blot analysis shows that GBE1 protein expression is decreased in the proband's explanted liver tissue. Beta-actin is used as a loading control. B) Progression of cardiomyopathy and heart failure after the liver transplant was shown by the increased thickness of a left ventricular posterior wall (LVPW) and decreased Ejection Fraction (EF%) measured by transthoracic echocardiogram.

**Clinical course:** Once the VUS (p.Ile694Asn) was determined to be disease-causing in both liver and heart, the transplant teams utilized this information to strategize clinical management. The proband was listed for a dual heart and liver transplant since both organs were involved and there is a known risk of cardiomyopathy progression and post-transplant mortality in patients who receive liver transplant alone [5,[9], [10], [11]]. He was placed on the liver transplant waitlist with a Pediatric End-Stage Liver Disease (PELD) score of 35 granted by the United Network of Organ Sharing (UNOS) exception policy, and the heart transplant waitlist with Heart Status 1B since an exception request was declined. Due to this denial of exceptional listing and scarcity of organ donation, he remained on both lists for approximately a year while his hepatic condition progressively worsened. His heart function remained relatively preserved. Once he developed profound liver failure with multiple episodes of variceal bleeding after a year of waiting for both organs, the liver transplant team decided to forgo the dual transplant since his heart listing status remained at low priority. The transplant team pursued a liver-alone transplant, knowing the 30–40% risk of heart failure post-liver transplant. Immediately after the transplant, he was infused with anti-IL2R (Basiliximab) to induce immunosuppression as a part of standard post-transplant care. The liver transplant was successful, and he was discharged from the hospital and returned home. Contrary to the results of the iPSC model, explanted liver tissue showed decreased GBE1 protein expression compared to healthy liver tissue, correlating with our iHep^GBE1-I694N^ findings (Fig. 4A). He was treated with standard immunosuppressants, including corticosteroids and tacrolimus. Corticosteroid was tapered off over 2 months, and he developed tacrolimus-induced hypertension, which was controlled with Amlodipine. Three months post-liver transplant, his cardiomyopathy progressed, evidenced by echocardiogram findings including an increasing thickness of left posterior ventricular wall and decreasing EF% (Fig. 4B). Five months post liver transplant, he developed septic shock without positive blood culture, likely due to a viral etiology, and acute heart failure with cardiovascular collapse. After entering acute heart failure status, he quickly progressed to multi-organ failure and passed away without the opportunity for a heart transplant.

## 4 Discussion

We present a case with suspected GSD4 in a one-year-old boy with an inconclusive genetic test. The sequencing of *GBE1* showed a known missense variant, which causes mild GBE deficiency, and a missense variant, p.Ile694Asn, of unknown clinical significance at the time of diagnosis [15,16]. Given the uncertainty of how the VUS influences the clinical course, we tested its disease causality and manifestation using a CRISPR/Cas9 genome-edited iPSCs *in vitro* system. We determined this missense variant reduced GBE1 enzyme expression and activity in both hepatocytes and cardiomyocytes. These findings supported a disease-causing genotype and disease phenotype in the liver and heart, which helped to guide the clinical practice for this ultra-rare disease.

Predictability of the clinical course was central to our case. GSD diagnosis was attainable through clinical assessment and histological examination of biopsies, but determining the subtype presented challenges, as GSD4 subtyping often relies on enzyme activities of biopsied tissues and prolonged clinical observation. Many patients with progressive hepatic subtypes face urgent liver transplantation needs within 2–3 years of diagnosis, underscoring the necessity for swift subtyping [3]; a diagnostic test that measures GBE1 tissue enzyme activity provides insight into disease severity and potential subtyping [28]. In this study, while liver GBE1 activity was low, heart biopsies did not yield sufficient tissue for assessment. Despite the significance of measuring GBE1 activity for GSD4 diagnosis, technical obstacles, such as ample tissue requirements, impede its routine application [8,12]. Our approach using iPSC-based modeling elucidated the correlation between GBE1 protein level, enzyme activity, and organ-specific manifestations. Leveraging CRISPR/Cas9-integrated iPSC differentiation techniques, we demonstrated the feasibility of studying GBE1 enzyme activity across various cell types and genotypes. This investigation offers a foundational proof of concept for mapping genotype-phenotype correlations in rare genetic disorders with broad clinical manifestations and therapeutic impact.

This study demonstrates how accurately iPSC-based modeling can predict multiple organ involvement in ultra-rare diseases with VUS and pathogenic variants and how valuable this modeling could be for personalized care of rare disease patients. Many protocols have been established to differentiate iPSCs into any cell type [29]*,* and iPSC-based modeling of disease and testing of novel variants is feasible as protocols for CRISPR/Cas9 genome editing are well established [17,30]. By identifying novel variants in rare diseases, inducing the novel variant in healthy iPSCs with CRISPR/Cas9 editing, and differentiating those cells to the desired cell types, clinicians and researchers garner a better understanding of how variants affect cells and can use that to guide patient care and future research.

Despite how promising iPSC-based modeling of rare diseases is, this system has some drawbacks. Culturing and growing iPSCs is time-intensive and expensive. Though it is possible to derive iPSCs from patient samples [29], generating and validating a research grade iPSC line costs $20,000–$35,000 [31,32]. From initial specimen collection to final characterization, it can take up to 9 months to generate iPSCs usable in a lab setting [31,32]. Additionally, once iPSCs have been generated they remain time-consuming to work with and expensive to maintain as they require daily medium changes, with expensive medium compared to other cell cultures [32,33].

Another limitation of iPSCs is that they do not differentiate to fully mature cells *in vitro* [29,34], indicating that differentiated iPSCs may not acquire all functions of target cells. However, despite our iHeps and iCMs showing features of immature hepatocytes and cardiomyocytes [[35], [36], [37]], these cells gained adequate functionality for our study. GBE1 is active *in utero,* in the fetus and placenta, and at birth and patients with GSD4 show GBE1 deficiency in these time frames [3,38,39]. Thus, testing the effects of the variant in *GBE1* in iHeps and iCMs provided an accurate evaluation of how the variant affected those cell types *in utero* and during infancy.

For additional remark, despite the initial plan of a dual transplant to mitigate cardiac mortality, the proband's heart transplant priority ranking remained low, and as a life-saving measure when his liver failed, we pursued a liver transplant alone. Against previously reported 60–70% survival probability after liver transplant, his cardiac comorbidities were rapidly exacerbated, and his quick descent into heart and multiple organ failure did not allow enough time for further intervention before he passed. It is unclear what his cardiac disease progression would have been in the absence of an additional clinical insult. Specifically, he was developing progressive hypertrophy post liver transplant. Although he was asymptomatic until his hospitalization, suggesting this was not progressive heart failure, his heart may already have been at risk for an additional clinical insult to tip him into acute heart failure. The dual heart-liver transplant remains a promising treatment option; however, the organ scarcity and the lack of exceptional listing in the UNOS-Heart Status will need to be addressed.

Regardless of iPSC modeling's limitations, it provides a novel and pragmatic model for identifying patients with specific genotypes requiring complex risk assessment for therapeutic decisions. This additional *in vitro* assessment supported our decision to prepare the patient for a dual liver and heart transplant since a solitary liver transplant would likely expose him to cardiac mortality risk. Because the dual liver and heart transplant at this age imposes tremendous difficulty on post-op care, confirming the subtype of GSD4 at the molecular genetic level was the critical factor for the transplant team to pursue the challenging surgical option. Despite not being able to offer the desired care in this instance, iPSC-based modeling has proven invaluable as a diagnostic and predictive tool for clinicians dealing with VUS in exceptionally rare diseases. The continued utilization of this model could pave the way for personalized medicine and improved treatment outcomes for rare disease patients, establishing iPSC-based modeling as a critical asset in the clinical arsenal for characterizing and managing complex, rare diseases.

## Funding

This work was supported by the 10.13039/100010327Cincinnati Children's Research Foundation, The Peter & Tommy Fund and the Colucci Family Foundation, NIH grant PSH grant (P30 DK078392).

## Previous presentations

A part of this manuscript was presented at the NASPGHAN annual meeting poster presentation.

## List of “human genes”

Symbol: GBE1.

Approved Name: 1,4-alpha-glucan branching enzyme 1.

Other Names: Glycogen branching enzyme 1, Andersen disease, glycogen storage disease IV.

Symbol: TNNT2.

Approved Name: troponin T2, cardiac type.

Other Names: Troponin T, CMPD2.

Symbol: HNF4A.

Approved Name: hepatocyte nuclear factor 4 alpha.

Other Names: NR2A1; HNF4.

## CRediT authorship contribution statement

**Chie Naito:** Data curation, Formal analysis, Investigation, Methodology, Writing – original draft. **Karis Kosar:** Data curation, Investigation, Visualization, Writing – original draft, Writing – review & editing. **Eriko Kishimoto:** Methodology, Resources. **Loren Pena:** Investigation. **Yilun Huang:** Methodology. **Kaili Hao:** Methodology. **Anas Bernieh:** Investigation. **Jennifer Kasten:** Investigation. **Chet Villa:** Investigation. **Priya Kishnani:** Formal analysis, Investigation, Resources. **Bali Deeksha:** Formal analysis, Investigation, Resources. **Mingxia Gu:** Investigation, Resources. **Akihiro Asai:** Conceptualization, Funding acquisition.

## Declaration of competing interest

All Authors disclose no conflicts of interest, including specific financial interests and relationships and affiliations relevant to the subject of our manuscript.

## Figures and Tables

**Figure:**
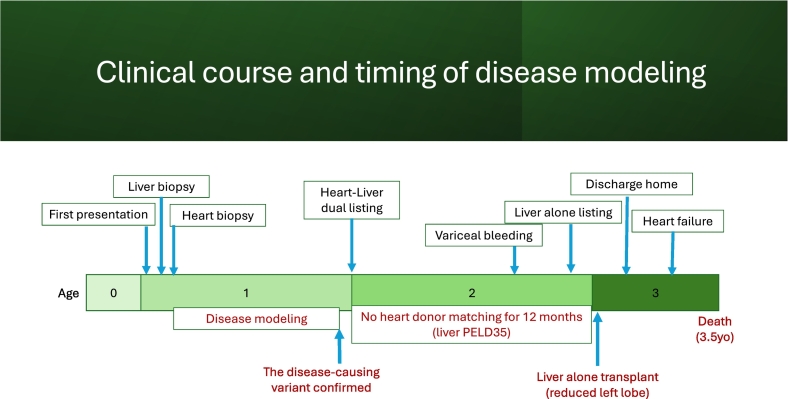


## Data Availability

Data will be made available on request.
